# Sequencing the pandemic: rapid and high-throughput processing and analysis of COVID-19 clinical samples for 21 ^st^ century public health

**DOI:** 10.12688/f1000research.28352.1

**Published:** 2021-01-26

**Authors:** Megan L. Folkerts, Darrin Lemmer, Ashlyn Pfeiffer, Danielle Vasquez, Chris French, Amber Jones, Marjorie Nguyen, Brendan Larsen, W. Tanner Porter, Krystal Sheridan, Jolene R. Bowers, David M. Engelthaler

**Affiliations:** 1 Pathogen Genomics Division, Translational Genomics Research Institute, Flagstaff, AZ, 86005, USA; 2 Department of Ecology and Evolutionary Biology, University of Arizona, Tucson, AZ, 85721, USA

**Keywords:** Genomic epidemiology, SARS-CoV2, targeted genomics, sequencing methods, phylogenetics

## Abstract

Genomic epidemiology has proven successful for real-time and retrospective monitoring of small and large-scale outbreaks. Here, we report two genomic sequencing and analysis strategies for rapid-turnaround or high-throughput processing of metagenomic samples. The rapid-turnaround method was designed to provide a quick phylogenetic snapshot of samples at the heart of active outbreaks, and has a total turnaround time of <48 hours from raw sample to analyzed data. The high-throughput method was designed for semi-retrospective data analysis, and is both cost effective and highly scalable. Though these methods were developed and utilized for the SARS-CoV-2 pandemic response in Arizona, U.S, and we envision their use for infectious disease epidemiology in the 21 ^st^ Century.

## Introduction

With the advent of rapid and inexpensive next-generation sequencing, genomic epidemiology has proven to be an invaluable resource for the elucidation of disease outbreaks. Extending beyond traditional shoe-leather approaches, rapid-turnaround sequencing methods have allowed researchers to quickly gain insight into the genetic nature of pathogens at the heart of active outbreaks ^1– 6^. By monitoring pathogen evolution over the course of an outbreak, large-scale genomics have the potential to allow for transmission mapping for infection control and prevention ^7, 8^, to distinguish independent cases from those part of active clusters ^9^, and to identify epidemiological patterns in time and space on both local and global scales ^7, 10– 12^.

The most recent example of this has been the collaborative genomic efforts mounted in response to the SARS-CoV-2 outbreak. Not long after the initial cases were identified, whole-genome sequencing quickly established the etiologic agent as a novel coronavirus ^13^, the origins of which have since been elucidated ^14^. Following the rapid spread of SARS-CoV-2, current next-generation technology and analysis pipelines allowed viral sequencing to take place on an unprecedented global scale, with collaborative consortia forming world-wide for the specific purpose of tracking and monitoring the pandemic ^15– 17^.

In most instances, including with the SARS-CoV-2 outbreak, genomic epidemiology has provided a retrospective view of pathogen spread and evolution well after the information is useful in the public health response to the outbreak ^1, 2^. Genomic epidemiology should guide contemporaneous outbreak control measures, but can only do so if the data are generated and interpreted in real-time quickly enough to inform a response ^18^. As technology has advanced, the potential exists to move beyond providing a retrospective genomic snapshot of an outbreak months after its occurrence, to providing actionable data in real-time for current outbreaks within hours after cases are identified ^4^. Real-time genomic tracking has already proven valuable in a number of instances, including the recent West-African Ebola outbreak ^3, 18^.

This is not to discredit the value of large-scale, retrospective studies. While rapid-turnaround genomics may prove essential for outbreak containment, retrospective studies will continue to be necessary to track pathogen evolution, gauge success of public health interventions, and to evaluate pathogen/host movement and behavior. With the recent SARS-CoV-2 pandemic, retrospective studies have so-far proven successful in identifying the timing and sources of outbreaks on a local ^19^ and global scale ^5, 20^, in evaluating the effectiveness of early interventions ^5^, and in identifying super-spreader events ^21^. Thus, in addition to real-time monitoring, high-throughput, cost-effective sequencing and analysis are needed to gain a better understanding of pandemics.

Here, we report two Illumina-based sequencing and analysis strategies for either real-time monitoring or large-scale, high-throughput targeted genomic sequencing of complex samples. Though these strategies were developed for use with the current SARS-CoV-2 pandemic, we envision their potential use in any situation in which a genomic response is needed.

## Methods

### Sample information

Remnant nasopharyngeal swab specimens or extracted RNA were obtained from, or received by, the TGen North Clinical Laboratory (TNCL) in Flagstaff, AZ. All samples had previously tested positive for SARS-CoV-2 by RT-PCR.

### RNA extraction

RNA was extracted using the MagMax Viral Pathogen II kit and a Kingfisher Flex automated liquid handler (ThermoFisher Scientific), with a DNase treatment incorporated to maximize viral RNA recovery from low viral-burden samples, defined as having an RT-PCR cycle-threshold (Ct) value above 33.0. These methods allowed for the rapid, scalable processing of a small number to hundreds of samples at once with minimal personnel, and prevented RNA extraction from becoming a bottleneck to overall throughput ( https://doi.org/10.17504/protocols.io.bnkhmct6). Remnant RNA was obtained from TNCL, and had been extracted following their FDA emergency use authorization protocol for the diagnosis of COVID-19.

### Targeted amplification

**Figure 1.**
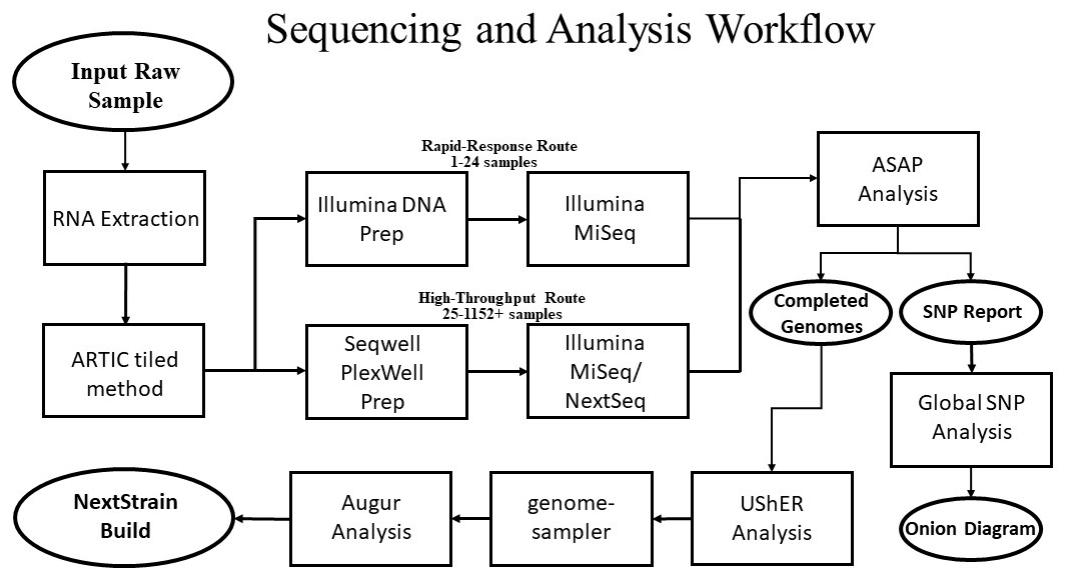
General workflow for the processing of complex samples. Basic workflow for processing of either high-throughput or rapid-response samples.

SARS-CoV2 RNA was amplified for both of the sequencing methods described below following the nCoV-2019 sequencing protocol V.1 ^22^ and using the ARTIC v3 primer set ^23^. Adapters were added to the resulting amplicons by one of the following means described below. The full sample processing workflow, starting with raw RNA and ending with deliverable data, is illustrated in Figure 1 for reference.

### Rapid-turnaround adapter addition and sequencing

For samples requiring immediate attention, e.g. those from patients potentially involved in an active outbreak, adapters were added with the DNA Prep kit (Illumina) as previously described ^24^. Amplicons were sequenced on the MiSeq platform, using a Nano 500 cycle kit with v2 chemistry (Illumina) ( https://doi.org/10.17504/protocols.io.bnnbmdan).

### High-throughput adapter addition and sequencing

In instances where retrospective data were needed from large numbers of samples, adapters were added with the plexWell384 kit (Seqwell). Samples were multiplexed in batches of 1,152 and sequenced on a NextSeq 550 with v2 chemistry and 150 X 150 bp reads (Illumina). When batch sizes were not large enough to fill a NextSeq run, samples were sequenced on a MiSeq, with V3 chemistry (Illumina) ( https://doi.org/10.17504/protocols.io.bnkimcue).

### Data processing and analysis

Virus genome consensus sequences were built using the Amplicon Sequencing Analysis Pipeline (ASAP) ^25, 26^. First, reads were adapter-trimmed using bbduk ^27^, and mapped to the Wuhan-Hu-1 genome ^28^ with bwa mem ^29^ using local alignment with soft-clipping. Bam alignment files were then processed to generate the consensus sequence and statistics on the quality of the assembly by the following: 1) Individual basecalls with a quality score below 20 were discarded. 2) Remaining basecalls at each position were tallied. 3) If coverage ≥10X and ≥80% of the read basecalls agreed, a consensus basecall was made. 4) If either of these parameters were not met, an ‘N’ consensus call was made. 5) Deletions within reads, as called during the alignment, were left out of the assembly, while gaps in coverage (usually the result of a missing amplicon) were denoted by lowercase ‘n’s. Only consensus genomes covering at least 90% of the reference genome with an average depth of ≥30X were used in subsequent analyses. Consensus genomes generated using these methods include those from Ladner *et al*. ^19^. Statistics reported for each sample included: total reads, number of reads aligned to reference, percent of reads aligned to reference, coverage breadth, average depth, and any SNPs and INDELs found in ≥10% of the reads at that position.

### Phylogenetic inference

Rapid phylogenetic inference was used when it was critical to rapidly answer two initial genomic epidemiological questions: i) are the samples in this set closely related, and ii) to what samples in the public database are these most closely related? To answer the former, SNP comparisons were made among a sample set of interest and output in text format directly from the ASAP results, foregoing the computational time of generating phylogenetic trees.

To answer the latter, a database of all SNPs in the GISAID global collection ^16^ was generated and periodically updated by downloading all the genomes and filtering them for quality and completeness, then aligning to the WuHan-Hu-1 reference to identify any variants, as described ^30^. The list of all variants and metadata for each sample were then put into a relational database for fast querying. The list of SNPs common to samples in a given set was then compared to this global SNP database using a custom script to determine how many GISAID genomes shared all or a subset of the SNPs from the set. A stacked Venn diagram to illustrate globally-shared SNPs was constructed using the statistical package “R” (Version 4.0.2) ^31^ and the “ggplot2” ^32^ and “ggforce” ^33^ R-packages.

For subsequent, more sophisticated phylogenetic analyses, phylogenetic trees were constructed in NextStrain ^17^, with genomes from GISAID subsampled by uploading our genomes of interest to the UCSC UShER (UShER: Ultrafast Sample placement on Existing tRee) tool ^34^, identifying relevant genomes (those most related to our genomes of interest), and further reducing that set of genomes when necessary with genome-sampler ^35^.

### Consent

Samples used were remnant, de-identified samples from a clinical diagnostics lab. No ethical approval was required for their inclusion in this study.

## Results

### High-throughput workflow

**Figure 2.**
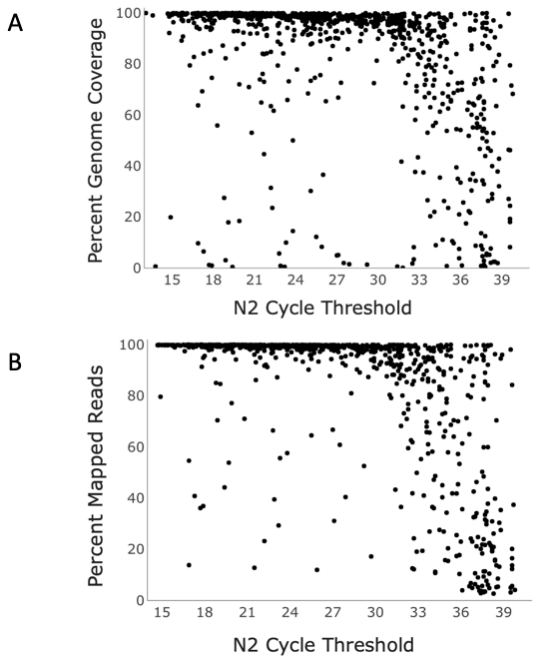
Sequencing outcomes of SARS-CoV2-positive samples processed using Seqwell’s plexWell method. **A**. Nucleocapsid-2 Ct value vs percent genome coverage and **B**. percent total reads mapped to SARS-CoV-2 for 897 SARS-CoV-2-positive samples sequenced using Seqwell’s plexWell 384 system.

For the Seqwell workflow, turnaround time from raw sample to sequence data was approximately 72 hours, and to phylogenetic inference approximately 10 hours, for 1,152 samples ( Table 1). Cost per sample was ~$50, which is comparable to similar sample prep methods ( Table 1) ^36^. Success rates, defined as the percentage of samples with greater than 90% genome coverage, were similar to other previously described methods ^36, 37^. Of a random subset of 897 samples analyzed, approximately 83% of samples with a Ct <33 yielded genomes with ≥90% breadth of coverage of the reference genome, i.e., a complete genome, with an average breadth of coverage of the reference genome of 91%. ( Figure 2, Table 2). Failure to obtain a complete genome when Ct was <33 was attributed to sample preparation error, sample degradation, or poor sequencing run metrics. Beyond a Ct of 33, success rate dropped drastically. Between Ct values of 33 and 35, complete genome success rate was ~41% ( Table 3), with an average breadth of coverage of 78%. Above a Ct of 35, ~18% of samples yielded a complete genome, and breadth of coverage dropped below 57% ( Table 2).

**Table 1.** Cost and labor analysis of two Illumina-based prep methods for complex sample processing. Workflow metrics for Illumina’s DNA prep method, and Seqwell’s plexWell method. * Listed is the number of samples that can be processed within the specified turnaround time, on a single sequencing run. This is not necessarily the upper limit of either processing system. ** Time from raw sample to analyzed data

Sequencing method	Samples able to be processed ^*^	Cost per Sample	Personnel Needed	Turnaround time ^**^
Illumina DNA Prep	24	$93.54	1	48hrs
Seqwell plexWell	1152	$50.55	4-6	82hrs

**Table 2.** Sequence metrics of SARS-CoV2-positive samples processed using Seqwell’s plexWell method. Average sequencing metrics for various RT-PCR cycle threshold values of samples sequenced using the plexWell 384 system, and either an Illumina MiSeq or an Illumina NextSeq550. *Ct value reported is that for the nucleocapsid-2 gene **Uniform depth of coverage was targeted for all samples

Average Cycle Threshold Value (n) ^*^	Average % aligned	Average % coverage	Average depth ^**^	% samples > 90% coverage
<20 (194)	96.64	92.36	3371.58	87.63
20-25 (210)	96.91	92.61	2848.11	85.24
25-30 (169)	95.67	91.11	2733.37	84.62
30-33 (110)	88.58	87.18	2113.09	74.55
33-35 (71)	74.68	77.85	1221.05	40.85
35-37 (52)	60.20	56.91	716.43	17.31
37+ (91)	38.28	52.71	576.09	18.68

**Table 3.** Sequence metrics for SARS-CoV2-positive samples processed using Illumina’s DNA Prep method. Average sequencing metrics for various RT-PCR cycle threshold values of samples sequenced using the Illumina DNA Prep system and an Illumina MiSeq. *Ct value reported is that for the nucleocapsid-2 gene **Uniform depth of coverage was targeted for all samples

Average Cycle Threshold Value (n) ^*^	Average % aligned	Average % coverage	Average depth ^**^	% samples > 90% coverage
<25 (6)	96.56	94.53	1503.00	83.33
25-30 (8)	91.92	87.60	1115.25	50%
30-37 (3)	56.46	62.83	851.21	0%

Uniform depth of genome coverage across samples was targeted when pooling samples for sequencing, but depth generally decreased as Ct increased. Average depth of coverage was approximately 2766X through a Ct of 33. Past 33, coverage dropped off sharply, with an average depth of 1221X between Ct values of 33-35. Between Cts of 35 and 37, coverage was 716X, and above a Ct of 37, coverage dropped to 576X ( Table 2).

Similarly, percent of total reads aligning to the SARS-CoV-2 reference genome decreased as Ct increased ( Table 2, Figure 2). Up to a Ct of 30, most (~96%) of reads mapped to SARS-CoV-2. From Cts of 30 to 35, this noticeably decreased, and continued to drop as Cts rose. Beyond a Ct of 37, average percentage of reads aligning dropped to 38%.

### Rapid-Turnaround Workflow

Turnaround time for the Illumina DNA prep method was significantly faster than the high-throughput plexWell system. It took less than 48 hours to go from raw sample to deliverable, analyzed data ( Table 1), and this time could potentially be reduced further by reducing cycle numbers per sequencing run.

Both the plexWell and DNA Prep methods use a tagmentation system for adapter addition, rather than a ligation-based approach. This results in adapters being added ~50 bp or more from the end of overlapping amplicons generated during gene-specific PCR. A second PCR step amplifies only the tagmented regions, resulting in final libraries of ~300bp. These approaches negate the need for primer trimming prior to alignment.

Generating consensus genomes and a SNP report from the sequence data, which takes approximately 15 minutes for a small (<=24 samples) dataset, quickly shows whether the samples are part of the same outbreak or transmission network. To quickly find a potential origin of a cluster or sample (assuming genomes in the public domain were collected prior to the sample(s) of interest), a global SNP database can be used. Constructing or updating a global genome SNP database takes several hours, but it can be done prior to sample sequencing (and regularly). Running the script to query the global SNP database for particular SNPs of interest takes mere seconds.

**Figure 3.**
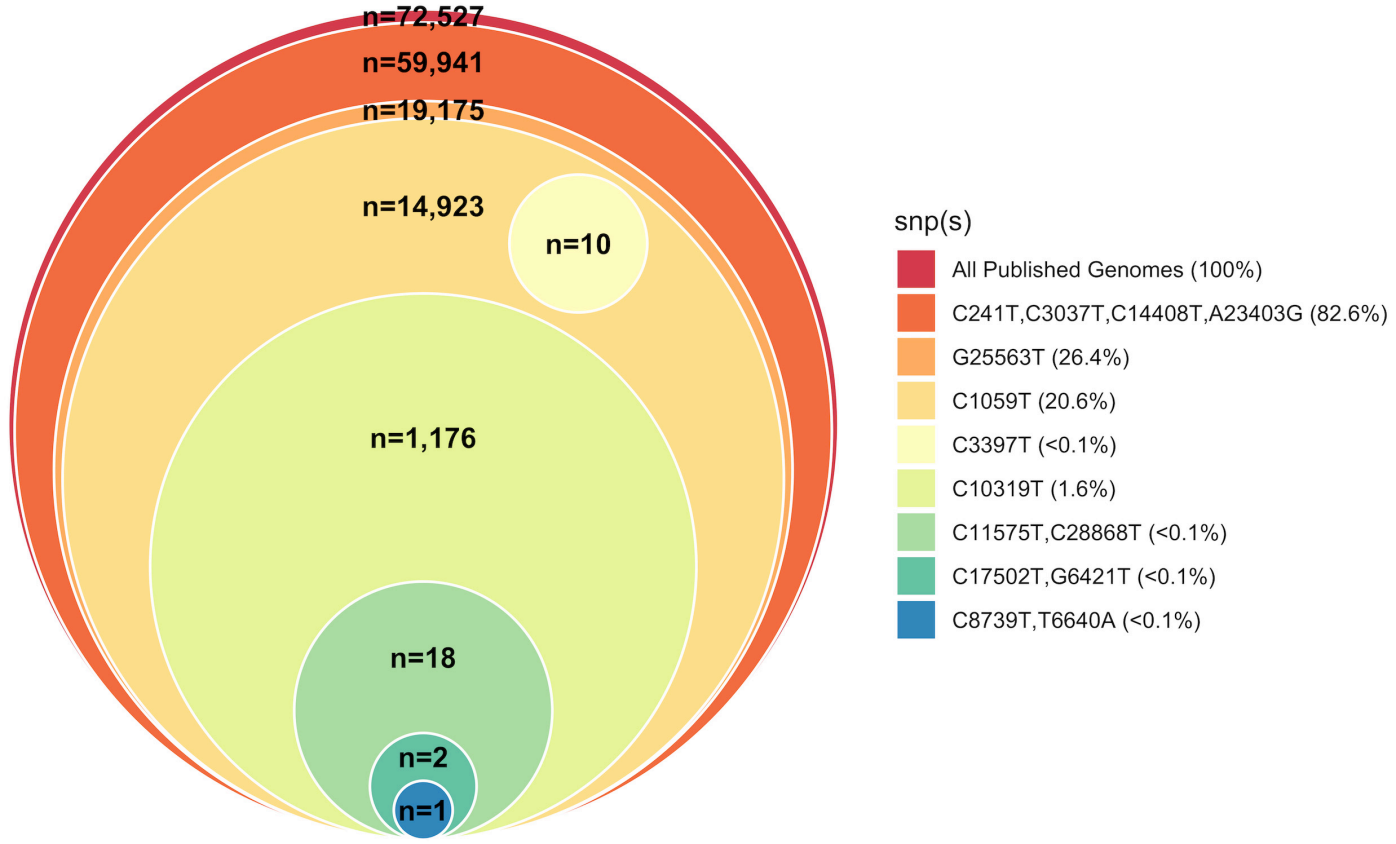
Phylogenetic relationship of SARS-CoV2-positive samples to Wuhan-Hu-1 reference strain. Stacked Venn “onion” diagram indicating the hierarchical nature of cluster-specific SNPs relative to the reference strain in global collection of SARS-CoV2 samples.

To rapidly visualize results of the global SNP database query, a stacked Venn diagram (aka, “onion diagram”) visually describes the hierarchical nature, i.e., the parsimony, of SNPs found in the SARS-CoV-2 genomes ( Figure 3), and is easily generated using the methods described above or an alternative tool.

Although fewer data are available from the Illumina DNA prep method, as it was primarily used to process 5 or fewer samples at a time, data suggest that it performed slightly worse than the Seqwell system, with odds of obtaining a complete genome dropping to 0 at Ct values greater than 30 ( Table 3).

## Discussion

The recent SARS-CoV-2 outbreak has highlighted the need for real-time sequencing and data analysis capacity in the face of active pandemics, as well as high-throughput sequencing and analysis strategies for comprehensive retrospective analysis and evaluation. We describe two different strategies that can be used in combination with Illumina platforms for the rapid or high-throughput interrogation of samples involved in disease outbreaks. Though the results reported here are specific to the SARS-CoV-2 outbreak, these methods could conceivably be modified and applied to many other situations in which a genomic epidemiological response is needed.

The Illumina DNA prep-SNP-comparison analysis method is effective in providing rapid sequence data and genomic epidemiology information for small numbers of samples (<48 hours from raw sample to rough phylogenetic placement). Though throughput is limited by both the number of available indices for multiplexing and the nature of the protocol itself, this method has the advantage of being scalable to small numbers of samples, and can be performed in the course of several hours for small sample subsets.

The Seqwell plexWell system provides a scalable, cost-effective, and high-throughput method of processing thousands of samples with minimal laboratory personnel ( Table 1). As the plexWell protocol calls for the pooling of samples at an initial step in the adapter tagmentation process, hundreds of samples were able to be taken through the later steps of the protocol by a single individual in an 8-hour timeframe. This, coupled with the availability of thousands of index combinations, allowed for a high-throughput, cost-effective means of processing large numbers of samples on a weekly basis with minimal laboratory personnel and infrastructure.

Typical analyses of virus genomes include phylogenetic tree construction to understand transmission patterns. Nextstrain ^17^ and GISAID ^16^ have been crucial to the SARS-CoV-2 scientific community for global and local epidemiologic understanding, and tools for smart subsampling (e.g. genome-sampler ^35^) are now necessary with the growth of the public databases. However, reconstructing phylogenies, especially paired with finding relevant subsets, takes time, and is often overkill for initial, time-sensitive public health needs. We employ a simple, rapid analysis method and visualization meant as a quick-look to determine relatedness among a sample set of interest and/or relatedness of a sample or set to the entire public database of genomes. Because of the novelty of SARS-CoV-2 and its low rate of recombination, merely comparing the low number of SNPs across samples without applying a phylogenetic model or program is often enough to answer initial questions about COVID-19 transmission, e.g. whether samples in a given set are closely related, and/or which samples in the global database are most closely related to a given sample set. Our SNP queries followed by generation of a stacked Venn diagram (onion diagram) offer a much faster alternative or antecedent to complete phylogenetic analysis. Other pathogens or situations where SNP numbers are expected to be very low may also benefit from these rapid analysis methods.

For retrospective studies, time can allow for more robust phylogenetic analyses including smart subsamplers ^35^, such as NextStrain ^17^ and other commonly used phylogenetic tools; however their employment can significantly add time to a rapid response. The UShER tool ^34^, which can rapidly place genomes onto an existing SARS-CoV-2 phylogenetic tree, can greatly speed-up the final analysis. Parsing the output of UShER generates a subset of public genomes that are phylogenetically close to the samples of interest. This reduces the input dataset to subsamplers such as genome-sampler ^35^, which significantly reduces the computation time for further subsampling based on geography and time, which in turn significantly reduces the computation time for a NextStrain analysis.

Each of the two methods have their limitations. We observed a reduced success rate of the rapid Illumina DNA Prep method over the high-throughput Seqwell system, as evidenced by both decreased overall breadth of coverage and decreased success of obtaining complete genomes at Ct values above 30. It should be noted, however, that rapid prep study was conducted on a limited sample set, using samples from active outbreak clusters that were shipped from long distances through varying ambient temperatures. Samples used to evaluate the Seqwell system were obtained locally and processed entirely in-house. This difference in handling, coupled with the sample size difference, may in part account for the differences in results in the two prep methods. Also, at ~$100/sample in reagent costs, the rapid Illumina DNA Prep method is less cost-effective ( Table 1). And though the turnaround for the protocol is ~1.5 days, faster sequencing is achievable through other methods, such as through the use of long-read Nanopore sequencing ^18, 36^. Nanopore technology, however, has the disadvantage of having a higher per-base error rate when compared to short-read sequencing methods ^18^, thus its lack of accuracy may outweigh any potential time savings, particularly in situations where relatively few nucleotide variants can radically alter phylogenetic placement such as for SARS-CoV-2. Also, the SNP-comparison analysis is rapid and robust because of the relatively low numbers of SNPs so far documented in the SARS-CoV-2 genome, due to the virus’s novelty, the lack of recombination, and the unmatched robustness of the global SARS-CoV-2 genome database. Though this method could be applied to other types of outbreaks, rapid, precise phylogenetic placement will rely on these same factors.

The high-throughput SeqWell prep system also has its drawbacks. The pooling strategy of the plexWell system prevents downscaling, thus small numbers of samples cannot be processed effectively with this system. The turnaround time of this method, when large sample numbers are processed, is not competitive ^36^. The majority of processing time is lost to the ARTIC portion of the protocol, however, and not specifically to the plexWell adapter addition. And though the combinatorial index system allows for thousands of samples to be multiplexed in a single sequencing run, the SARS pandemic has demonstrated that even this may not be sufficient to meet the data challenges presented by expansive disease outbreaks.

Despite overwhelming benefits of employing next generation technological advances in real-time during a public health emergency, challenges remain when using genomic epidemiology as a means of pandemic control and monitoring. It has been demonstrated that genomics are not, in and of themselves, sufficient to completely elucidate the mechanisms and transmission of all pathogen-transmitted disease, particularly when asymptomatic and mild infections are known to play a role in transmission ^38^ but are less likely to be identified and subsequently sequenced. Thus, the integration of genomic and traditional epidemiology is paramount to the success of this 21 ^st^ century public health capability.

## Data availability

### Underlying data

Zenodo: Raw sequencing metrics from two different prep methods for obtaining SARS-CoV2 genomes, http://doi.org/10.5281/zenodo.4309900 ^39^.

This project contains the following underlying data:

- Sequencing_metrics_IlluminaDNAprep.xlsx- data used for generation of Table 3
- Sequencing_metrics_Seqwellprep.xlsx- data used for generation of Figure 2 and Table 2

Data are available under the terms of the Creative Commons Attribution 4.0 International license (CC-BY 4.0).
