# Supplementary material for: Methods for sequencing the pandemic: benefits of rapid or high-throughput processing
Source: F1000Res. 2022 Feb 21;10:ISCB Comm J-48. Originally published 2021 Jan 26. [Version 2] doi: 10.12688/f1000research.28352.2 (PMC8921685; doi:10.12688/f1000research.28352.2)
Supplement: Peer review history

# Peer review: Reviewer response for version 2

**DOI:** 10.5256/f1000research.120334.r124549

**Published:** 2022-03-14

**Authors:** Bronwyn L. MacInnis

**Affiliations:** 1 Broad Institute of Harvard and MIT, Cambridge, MA, USA

The authors have addressed my comments in this version of the manuscript, congratulations on a good paper.

Is the rationale for developing the new method (or application) clearly explained?

Yes

Is the description of the method technically sound?

Yes

Are the conclusions about the method and its performance adequately supported by the findings presented in the article?

Yes

If any results are presented, are all the source data underlying the results available to ensure full reproducibility?

Yes

Are sufficient details provided to allow replication of the method development and its use by others?

Yes

Reviewer Expertise:

Virology, Genomics, Epidemiology

I confirm that I have read this submission and believe that I have an appropriate level of expertise to confirm that it is of an acceptable scientific standard.

# Peer review: Reviewer response for version 2

**DOI:** 10.5256/f1000research.120334.r124550

**Published:** 2022-03-07

**Authors:** Keith Crandall

**Affiliations:** 1 Computational Biology Institute, The George Washington University, Washington, DC, USA; 2 Department of Biostatistics & Bioinformatics, The George Washington University, Washington, DC, USA

The authors have responded well to my previous critiques. I think the paper now provides useful information for sequencing methods (read depth, coverage, etc.) and includes some statistics to justify some of the conclusions in the paper.

Is the rationale for developing the new method (or application) clearly explained?

Yes

Is the description of the method technically sound?

Yes

Are the conclusions about the method and its performance adequately supported by the findings presented in the article?

Partly

If any results are presented, are all the source data underlying the results available to ensure full reproducibility?

No

Are sufficient details provided to allow replication of the method development and its use by others?

Partly

Reviewer Expertise:

phylogenetics, bioinformatics, computational biology, infectious disease

I confirm that I have read this submission and believe that I have an appropriate level of expertise to confirm that it is of an acceptable scientific standard.

# Peer review: Reviewer response for version 1

**DOI:** 10.5256/f1000research.31361.r92632

**Published:** 2021-09-21

**Authors:** Keith Crandall

**Affiliations:** 1 Computational Biology Institute, The George Washington University, Washington, DC, USA; 2 Department of Biostatistics & Bioinformatics, The George Washington University, Washington, DC, USA

This study proposes a reasonable approach to rapid assessment of viral diversity for SARS-CoV-2 taking advantage of the latest in Illumina sequencing kits and associated prep protocols as well as open source data and tools for downstream analyses. There are no statistics involved and therefore users have no idea how reliable any result might be coming out of such an approach. I would personally prefer public health decisions be based on accurate as well as rapid information. This paper speaks to rapid, but does not speak to accurate. Additionally, the paper describes some methodology that is unique to this work, but the authors do not provide any access to these crucial components. Therefore, as presented, the work seems to not be repeatable. This would need to be fixed before indexing. I have detailed these issues below.

 One of the biggest issues we have when consulting with individuals or groups interested in SARS-CoV-2 sequencing is deciding on read quantity. The methods discusses 'coverage' for confidence in genome assembly, but there is no discussion at all on the number of reads targeted for sequencing. What is the target number of reads for the SNP analysis versus the whole genome analysis? Can you provide power calculations for identifying SNPs based on read coverage? Quality of assembled genomes based on read coverage? I think such analyses would be particularly helpful to individuals trying to implement this strategy in their own labs.

 Is there a link to the 'global SNP database'?  Is this an open access resource? If so, you should provide a link to it. If not, then the paper is not particularly useful because the work is not replicable.

 Is the 'custom script' for SNP identification available? This should also be available via something like GitHub for reproducibility.

 It's too bad the authors didn't go through the effort of consenting the sample donors and including clinical data. The genomic information is highly useful (as outlined in the paper), but the genomic information COUPLED with the EHR and epidemiological data would be infinitely more useful.

 There don't seem to be any statistics associated with 'transmission network' assignment. How accurate is this inference and how is a user supposed to know about the accuracy of such an assignment?

 Similarly, there are no statistics associated with the phylogenetic placement of genomes. What is the confidence a user would have in such placement?

Is the rationale for developing the new method (or application) clearly explained?

Yes

Is the description of the method technically sound?

Yes

Are the conclusions about the method and its performance adequately supported by the findings presented in the article?

Partly

If any results are presented, are all the source data underlying the results available to ensure full reproducibility?

No

Are sufficient details provided to allow replication of the method development and its use by others?

Partly

Reviewer Expertise:

phylogenetics, bioinformatics, computational biology, infectious disease

I confirm that I have read this submission and believe that I have an appropriate level of expertise to state that I do not consider it to be of an acceptable scientific standard, for reasons outlined above.

# Response

**Published:** 2022-01-14

**Authors:** Megan Folkerts

**Affiliations:** Translational Genomics Research Institute- North, USA

**Dr. Crandall,**

 We want to thank you for taking the time to review our paper and provide valuable insight and comments aimed at strengthening our manuscript. We have carefully considered the comments, and have done our best to address each of them. We hope that the revised version of this manuscript addresses the concerns you voiced, and now meets your standards. The authors welcome further constructive criticism.

 Attached is a point-by-point response to both reviews. Changes made to the manuscript are clearly stated, with line numbers included for your quick reference.

 Sincerely,

 Megan Folkerts, MS

mfolkerts@tgen.org



Translational Genomics Research Institute, North









**Response to Reviewer 2 (Dr. Keith Crandall)**







**Comment 1: **There are no statistics involved.



**Response: **We agree that this was an oversight, even for a methods paper. All sequencing metrics are now reported in terms of mean, standard deviation, and 95% confidence interval.



**Comment 2: **There is no discussion at all on the number of reads targeted for sequencing.



**Response: **Thank you for this feedback. We have added read counts to all sequencing data metrics within the manuscript body. This is reported in the following paragraphs:



Lines 217-229: The number of reads targeted per sample for the high-throughput method was approximately 348,000, considering the limitation of the number of index combinations (i.e., samples per run) that were available at the time. In practice, this varied considerably. The average read count for samples using the plexWell method was 411,375, with 393,925[MOU2] being the average number of reads that aligned to the WuHan-Hu-1 reference strain. Below a Ct of 33, average aligned reads was 475,310 (SD 260292, 95%CI 455789-494831) per sample. Between Cts of 33 and 35, the average reads aligned was 204,234 (SD 175947, 95%CI 163308-245160) per sample. Above 35, read counts dropped off sharply, with a mean of 99,388 reads aligning (SD 188499, 95%CI 68493-130283) per sample. Note that all samples were put through an equimolar pooling step by the plexWell system. This suggests this pooling is not entirely effective when Cts vary considerably in a given sample set. This was confirmed through Kruskal Wallis on the total (unaligned) read counts in each of the above-mentioned groups (p <0.001)





Lines 289-296: A minimum of 42,000 reads were targeted for the Illumina DNA prep method, but as sample sizes were small for the outbreak in question, the average aligned reads per sample was much higher. For samples with Cts below 25, the average number of reads aligned to the reference was 275,258.7 (SD 136,816.2, 95%CI 165,784.9-384732.4). Between Cts of 25 and 30, average reads aligned was 200,810 (SD 86,999.43, 95%CI 140,523.6-261096.4). Below a CT of 30, average aligned reads dropped to 116,888 (SD 275257.7, 95%CI 53,002.2-180,773.8). Kruskal-Wallis revealed no significant difference between average aligned reads between these groups (P= 0.08).





**Comment 3: **What is the target number of reads for the SNP analysis vs the WGS analysis?



**Response: **The SNP analysis was run concurrently with the WGS analysis, and the read depth targeted for the SNP analysis is the same as that of the WGS analysis. Targeted read depth, and achieved read depth, has been added to the manuscript (see response to comment 2). A previously published study reported a depth of 10 reads sufficient for SNP calling of SARS-CoV2 genomes (Ladner et al 2020). In all instances, SNPs called using the methods published here far exceed this threshold. Average read depth per sample can be found in the supplementary data.





**Comment 4: **Can you provide power calculations for identifying SNPs based on read coverage?



**Response: **We agree that providing power calculations for SNPs based on read coverage is critical for phylogenetic analyses. The purpose of this paper is simply to report sequencing and analysis methods for the purpose of generating data that can then be fed into more thorough phylogenetic analyses, as we have done with Ladner et al 2020. This is reflected in the following:



Lines 122-123: Consensus genomes generated using these methods include those from Ladner *et al*. ^19^ . and are similar to those in Peng et al. ^43^ and Stoddard et al. ^44^





**Comment 5: **What is the quality of assembled genomes based on read coverage?



**Response: **Average depth of coverage, as it correlates to CT value and percent genome coverage, is summarized in Table 2 and 3. Average depth by sample across the same metrics is available in the Supplementary Methods.





**Comment 6: **Is there a link to the global SNP database?



**Response: **The global SNP database, which contains data directly downloaded from GISAID, is not able to be shared via secondary publications, as per GISAID’s data sharing agreement. The contents of the database are described, with one table generated using the Chan et al. ^35^ analysis on genomes downloaded from GISAID, and the other table generated directly from GISAID data which is available for public download from GISAID’s website, which is linked in the reference section. This is addressed via the following:



Lines 138-143: The format of this database was simply one table of SNPs, as generated by Chan et al. ^35^, with columns for GISAID ID, position, reference base, and variant base, and a second table of metadata which contained the exact data fields, as downloaded from GISAID. The two tables were linked by the GISAID ID number. The GISAID database cannot be shared via secondary publications as per the database access agreement, however the database is available for download by the public from GISAID’s website ^16^.





**Comment 7: **Is the custom script for SNP identification available?



**Response: **The custom script consisted of a simple set of commands, described in detail in the following so that they can be reproduced:



Lines 143-147: The list of SNPs common to samples in a given set was then compared to this global SNP database by first querying the number of global genomes containing each of the individual SNPs, sorting them from most common to least common, then further querying for number of global genomes containing combinations of SNPs by adding in each successive SNP in turn.





**Comment 8: **Genomic information coupled with the epidemiological data would be infinitely more useful



**Response: **We completely support this comment. However, this was not done here as it exceeded the purpose of this paper, which was to provide methods for rapid and high-throughput sample processing and initial phylogenetic placement of genomes. We agree that obtaining patient metadata can provide for a more robust analysis and meaningful interpretation for a more complete epidemiological picture, and in our publications where that is the goal, we make every effort to arrange this.





**Comment 9: **There don’t seem to be any statistics associated with the “transmission network”



**Response: **Thank you for this feedback. This language was changed to reflect the scope of the paper. We were not conducting full transmission analyses (as we overstated in the first draft), but rather, were using the obtained genomic data to guide field epidemiology. Current terminology reflects this, as per:



Lines 260-262: Generating consensus genomes and a SNP report from the sequence data, which takes approximately 15 minutes for a small (<=24 samples) dataset, quickly shows whether the samples are part of the same outbreak.





**Comment 10: **There are no statistics associated with the phylogenetic placement of genomes.



**Response: **This is correct. We have cited our previous phylogenetic analyses and other published works that have used and described similar methods. The purpose of this manuscript is primarily to report methods of generating data that can be fed into more complex phylogenetic analyses. We agree that statistics associated with placement are critical, and we have detailed complete phylogenetic analysis of SARS-CoV-2 genomes in Ladner et al. 2020, which follows the published methods of Bolyen et al 2020. Detailed phylogenetic analyses methods and results are not included here, as we felt that this was not part of this study. We have indicated this in the following:



Lines 336-340: However, reconstructing phylogenies, especially paired with finding relevant subsets, takes time, and is often overkill for initial, time-sensitive public health needs. We employ a simple, rapid analysis method and visualization meant as a quick-look to determine relatedness among a sample set of interest and/or relatedness of a sample or set to the entire public database of genomes.



Lines 349-351: This is not meant to replace more thorough phylogenetic analysis, but rather, to provide a quick genomic snapshot that can inform a public health ground response

# Peer review: Reviewer response for version 1

**DOI:** 10.5256/f1000research.31361.r78623

**Published:** 2021-03-16

**Authors:** Bronwyn L. MacInnis

**Affiliations:** 1 Broad Institute of Harvard and MIT, Cambridge, MA, USA

This is a methodologically sound study describing complementary approaches for generating and analyzing SARS-CoV-2 genomic data. The workflows address two common use cases: rapid turnaround of a relatively small number of samples, i.e. for cluster investigation, and larger scale but less urgently time sensitive sequencing. 

 The approach described here is sound and there is sufficient detail to follow the methodology. 

 My main issue with the manuscript is that it is not clear what the novel contribution is, since each of the approaches are previously described, using commercial and published methods. However it is nonetheless helpful to have the workflows mapped out as they are here, and to benchmark the differences between the approaches. This could be particularly helpful to the many labs now attempting viral/SARS-CoV-2 sequencing for the first time. 

 I would also advise that the focus on retrospective sequencing limits the potential value of the larger scale approach. It could equally be relevant for larger scale surveillance sequencing, i.e. for variants of concern, real time (or near real time). 

 I would like to see the costs described in more detail, and in relative terms, since costs of consumables and effort can vary widely across contexts. Please clarify what is included in costs in more detail, whether costs include effort, what the impact of effort is.

 Personally I find the title to be overstated for the content of the manuscript. I would suggest a title that more clearly conveys that this is a methods paper. In particular I would delete the prefix "Sequencing the pandemic", as this suggests something being done at a global scale, and the "21st century public health" as there is no direct application to public health. 

 Finally, although this study clearly focuses on lab methods for sequencing, given the framing I would like to see it acknowledge that currently many of the delays in SARS-CoV-2 sequencing are not in the sample preparation and sequencing steps, rather in processes up and downstream of sequencing.

Is the rationale for developing the new method (or application) clearly explained?

Yes

Is the description of the method technically sound?

Yes

Are the conclusions about the method and its performance adequately supported by the findings presented in the article?

Yes

If any results are presented, are all the source data underlying the results available to ensure full reproducibility?

Yes

Are sufficient details provided to allow replication of the method development and its use by others?

Yes

Reviewer Expertise:

Virology, Genomics, Epidemiology

I confirm that I have read this submission and believe that I have an appropriate level of expertise to confirm that it is of an acceptable scientific standard, however I have significant reservations, as outlined above.

# Response

**Published:** 2022-01-14

**Authors:** Megan Folkerts

**Affiliations:** Translational Genomics Research Institute- North, USA

**Dr. MacInnis,**

 We want to thank you for taking the time to review our paper and provide valuable insight and comments aimed at strengthening our manuscript. We have carefully considered the comments, and have done our best to address each of them. We hope that the revised version of this manuscript addresses the concerns you voiced, and now meets your standards. The authors welcome further constructive criticism.

 Attached is a point-by-point response to both reviews. Changes made to the manuscript are clearly stated, with line numbers included for your quick reference.

 Sincerely,

 Megan Folkerts, MS

mfolkerts@tgen.org



Translational Genomics Research Institute, North









**Response to Reviewer 1 (Dr. Bronwyn MacInnis)**





**Comment 1: **It is not clear what the novel contribution is



**Response: **We appreciate this valuable feedback and hope we have provided some clarity. This manuscript seeks to provide guidance to labs seeking to undertake rapid and or high throughput sequencing, and it reports the first use of the SeqWell plexWell system for use in conjunction with the commonly used ARTIC primer system. This novel method required few laboratory personnel and minimal equipment, and allowed for the rapid sequencing of thousands of samples per week, a novel pace for such a complex procedure. The manuscript has been reworded to highlight these developments. Changes mentioned here are reflected in the following lines of the manuscript:



Abstract: The high-throughput method, first reported here for SARS-CoV2,

Lines 45-46: The plexWell method is a novel means of sequencing adapter addition to facilitate high-throughput sample processing.

Lines 315-316: Here, we report the first use of plexWell for post-amplification adapter addition for whole genome sequencing of SARS-CoV2.

Lines: 321-325: With just two full-time staff members devoted to sequencing, and 2-4 part-time staff responsible for arraying samples prior to the ARTIC/plexWell protocol, 1152 samples were able to be successfully processed and sequenced each week. Thus, the number of genomes completed per full-time (40 hour) employee per week is 384, a novel pace for what is normally a lengthy protocol





**Comment 2: **Focus on retrospective sequencing limits the potential value of the larger scale approach.



**Response: **We agree with this comment; however, sequencing was not merely done retrospectively. All data shown for the rapid-turnaround method were done in real-time, and data were delivered fast enough to inform a public health response. The manuscript has been edited to make this clear via the following (which includes a citation of the relevant MMWR report for this study).



Lines 352-355: The data presented here using the rapid-turnaround method were obtained from patients who were part of an active SARS-CoV2 outbreak, and the resulting phylogenetic placements were successfully used to inform public health efforts to determine the potential origin of the outbreak, and to prevent further spread ^45^.





**Comment 3: **I would like to see the costs described in more detail.



**Response: **We have now provided a breakdown of sequencing costs in the supplementary methods. This includes the manufacturer’s reagent pricing, and represents the relative cost to the user minus any institution-specific discounts or advantages.



**Comment 4: **I find the title to be overstated



**Response:** We now recognize that the title overstated the scope of this methods paper. We have changed the title to more closely represent the content of the publication.



**Comment 5: **I would like to see it acknowledged that currently many of the delays in SARS-CoV2 sequencing are not in the sample preparation and sequencing steps, rather in the processes up and downstream of sequencing.



**Response: **We agree that delays aren’t just in sequencing, and have acknowledged and described the other areas of potential delay in the manuscript, as per:



Lines 398-407: This paper highlights the potential for rapid turnout of genomic data from epidemiological samples, giving genomics the potential to become invaluable in pandemic response. It is worth noting, however, that sequencing isn’t the only potential bottleneck in the process. For data to be delivered in real-time, coordination between testing sites, sample delivery, and sequencing laboratories must be robust, so that preparation for sequencing can begin shortly after a sample has tested positive for the pathogen of interest. Failures anywhere in this chain can lead to significant delays, which negates the utility of a real-time sequencing pipeline for investigation. Thus, in addition to improvements in the described sequencing and analysis pipeline, coordinating the efforts of testing sites, public health partners, and sequencing laboratories is critical for this technology to be most effective.
